# Echocardiographic parameters predicting spontaneous closure of ductus arteriosus in preterm infants

**DOI:** 10.3389/fped.2023.1198936

**Published:** 2023-06-15

**Authors:** Minyu He, Zhengchun Yang, Tian Gan, Jing Tang, Suzhen Ran, Kun Zhang

**Affiliations:** ^1^ Department of Ultrasound, Chongqing Health Center for Women and Children, Chongqing, China; ^2^ Department of Ultrasound, Women and Children's Hospital of Chongqing Medical University, Chongqing, China

**Keywords:** patent ductus arteriosus, infant, premature, echocardiography, congenital heart disease

## Abstract

**Objective:**

To evaluate the value of echocardiographic parameters in predicting early spontaneous closure of ductus arteriosus in premature infants.

**Methods:**

222 premature infants admitted to the neonatal ward of our hospital were selected, and patent ductus arteriosus was detected by echocardiography 48 h after birth. On the 7th day, whether the ductus arteriosus was closed naturally in this cohort was observed. The infants whose ductus arteriosus were not closed were identified as the PDA group (*n* = 109), and the other infants were included in the control group (*n* = 113). The echocardiographic parameters of the two groups of premature infants at 48 h after birth were single-factor statistically and Pearson correlation analyzed, and the parameters with statistically significant differences in single-factor analyzed were selected for multivariate logistic stepwise regression analysis.

**Results:**

The ductus arteriosus shunt velocity and the pressure difference between the descending aorta and the pulmonary artery (ΔPs) in the PDA group were lower than those in the control group (*P* < 0.05). The pulmonary artery pressure (PASP) in the PDA group was higher than that in the control group (*P* < 0.05). According to the multivariate logistic stepwise regression analysis, only the maximum shunt velocity of ductus arteriosus was correlated with early spontaneous closure of ductus arteriosus in 48 h first echocardiographic parameters (*P* = 0.049). The receiver operating characteristic (ROC) curve indicates the optimal critical point of echocardiographic ductus arteriosus shunt velocity in premature infants 48 h after birth was 1.165 m/s.

**Conclusion:**

Echocardiographic parameters are of great value in predicting the early spontaneous closure of ductus arteriosus in premature infants. In particular, the ductus arteriosus shunt velocity is correlated with the early spontaneous closure of ductus arteriosus.

## Introduction

In recent years, the incidence of premature infants has continued to rise, accounting for about 6%–12% of newborns (1). At the same time, premature infants are also the main group of neonatal intensive care (1). Patent ductus arteriosus (PDA) is one of the common congenital heart diseases in premature infants, accounting for about 10%–15% of congenital heart disease. The incidence of PDA in premature infants is higher than in full-term infants, and its incidence is negatively correlated with gestational age (1). Anatomically, the ductus arteriosus (DA) is the vascular structure connecting the descending aorta and the left pulmonary artery (2). When a newborn breathes for the first time, the lungs begin to function, and a series of changes occur in the blood circulation system. For instance, the resistance of pulmonary circulation starts to decrease, the pulmonary artery pressure (PASP) decreases, and the aortic systolic blood pressure (AOSP) increases. Consequently, the blood flowing through the ductus arteriosus decreases gradually, and the ductus arteriosus is functionally closed. Hemodynamically significant PDA (hsPDA) is associated with poor outcomes in preterm infants. Long-term exposure to hsPDA can lead to pulmonary circulation overload and systemic hypoperfusion, and increase pulmonary hemorrhage, bronchopulmonary dysplasia (BPD), necrotizing enterocolitis (NEC), acute kidney injury, periventricular white matter Malacia, intraventricular hemorrhage (intraventricular hemorrhage, IVH) and risk of death. Currently, there is no consensus on clinical and ultrasound standards for the treatment of PDA. The definition of hsPDA requires clinical manifestations combined with ultrasound judgment.

Functional closure of ductus arteriosus usually occurs within 48 h after birth in full-term infants (3). However, there is a high incidence of PDA in premature infants. When the ductus arteriosus is continuously open, the pulmonary blood flow increases and the systemic blood flow decreases, which could induce heart failure, bronchial dysplasia, pulmonary hemorrhage, intraventricular hemorrhage, abnormal cerebral perfusion, necrotizing enterocolitis, and other complications in premature infants (4), and in most severe cases can lead to death (5). Therefore, it is very important to take early intervention to induce the closure of ductus arteriosus. However, since ductus arteriosus can be closed spontaneously in most cases, it is also important to reduce unnecessary clinical interventions. At present, there are few reports about using a non-invasive method to predict spontaneous closure of ductus arteriosus in premature infants. Some scholars have suggested that over-active drug treatment cannot improve the short-term and long-term prognosis of premature infants with PDA (6). Studies of (1, 7) have shown that the best way to evaluate an unclosed ductus arteriosus is to measure its size and shunt velocity. The study of (8) found that the measurement of ductus arteriosus diameter on the first day after birth had high sensitivity and specificity in predicting the closure of ductus arteriosus. However, most of the studies are univariate analysis.

Previous literature has shown that the ultrasonic diagnostic criteria for hsPDA may include: ductus arteriosus diameter ≥1.5 mm, LA:AO ≥1.4, changes in mitral valve E/A value (indicators for assessing left ventricular diastolic function), etc. However, the measurement of these indicators depends on the left ventricular preload, and different sonographers will also affect the results. In addition, aortic diastolic pressure decreases when the ductus arteriosus shunts heavily from left to right. Descending blood flow from the descending aorta during systole, retrograde “steal blood” into the ductus and into the pulmonary arteries during diastole, resulting in relatively hypo perfused systemic arteries, including the cerebral hemispheres and gut, negatively affects preterm infants. At present, there is no conclusion about which population may benefit from PDA intervention. Therefore, early prediction of whether the ductus arteriosus can be closed naturally is of great significance for the need for early intervention in clinical intervention. The purpose of this study was to investigate the value of the first echocardiographic parameters 48 h after birth in predicting the early spontaneous closure of ductus arteriosus in premature infants.

## Methods

### Research object and grouping

**Figure 1:**
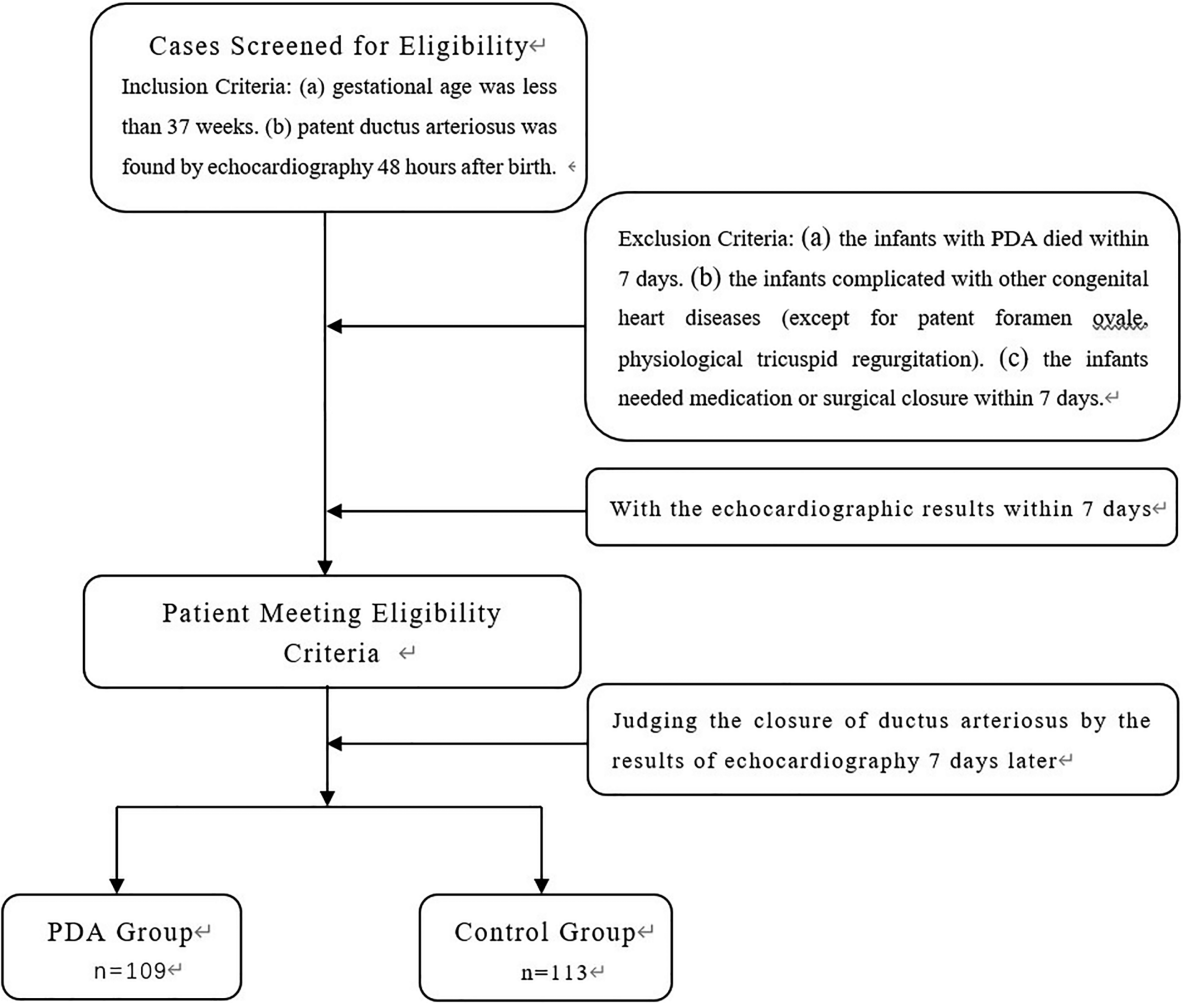
Flowchart of patient screening.

A total of 222 premature infants admitted to our neonatal ward from January 2017 to December 2022 were selected for the study. The inclusion criteria are: (a) gestational age was less than 37 weeks, and (b) patent ductus arteriosus was found by echocardiography 48 h after birth. The exclusion criteria are that the infants with PDA died within 7 days or were complicated with other congenital heart diseases (except for patent foramen ovale, physiological tricuspid regurgitation) or needed medication or surgical closure within 7 days. The echocardiographic results of these premature infants were observed on the 7th day. The detailed flow chart for patient selection is illustrated in Figure 1. 109 cases (56 males) whose ductus arteriosus was still open on the 7th day after birth were identified as the PDA group. The gestational age was 25.1–36.6 weeks, with an average of 31.72 ± 3.11 weeks; 113 cases (66 males) with spontaneous closure of ductus arteriosus within 7 days were treated as the control group. The gestational age was 26.5–36.5 weeks, with an average of 33.30 ± 2.48 weeks.

Among the 222 cases, 113 were premature infants whose ductus arteriosus closed naturally after 7 days: 88 cases had good clinical indicators and were discharged from the hospital; 25 cases continued to be hospitalized for observation due to neonatal pneumonia, neonatal jaundice, and substandard weight. 109 cases of premature infants whose ductus arteriosus did not close naturally after 7 days: 77 cases were discharged with good clinical indicators and were followed up with echocardiography; 30 cases continued to be hospitalized for observation due to neonatal pneumonia, neonatal jaundice, and substandard weight; 2 cases of premature infants complicated with NEC surgical treatment.

### Ultrasonography instruments and the methods

The GE Vivid i color Doppler ultrasonography, cardiac 6S probe and frequency 3∼7 MHz were used in the study. During the ultrasonography examination sessions, the preterm infants were made to lie on their backs. The measurement parameters included: (a) the aortic root diameter (AOD) and the left atrial end-systolic diameter (LAD) were measured on the parasternal long axis view, and LAD/AOD was calculated; (b) the left ventricular ejection fraction (LVEF), and fractional shortening (FS) were measured by M-mode echocardiography; (c) the tricuspid regurgitation area was measured in apical four-chamber view; and (d) the DA was observed in short axis view or suprasternal view, where the direction of the blood shunt was recorded, and the DA diameter of the DA shunt velocity were measured.

According to the simplified Bernoulli equation, the pressure difference between descending aorta and the pulmonary artery (ΔPs = 4 V_DA_^2^, V_DA_:DA shunt velocity) was calculated, and the pulmonary artery systolic pressure (PASP) was calculated. PASP = aortic systolic blood pressure (AOSP)—ΔPs. When there was no left ventricular outflow tract and aortic stenosis, the brachial artery systolic blood pressure, which is similar to AOSP, was used instead. The width of the foramen ovale was measured on a subxiphoid two-chamber view under the xiphoid process. All the images and data were collected and measured by two ultrasound physicians. Three cardiac cycles were measured and the average value was calculated.

### Statistical analysis

The SPSS25.0 software was used for statistical analysis. Measurement data was expressed in $\overset{-}{x}$ ± s. The categorical variable was expressed in percentage. An Independent sample *t*-test was used to compare the parameters of the ultrasound data collected 48 h after birth between the PDA groups and the control group. The categorical variable was compared by *χ*^2^ test, and a *P* < 0.05 was considered statistically significant. The parameters with statistical significance were selected for multivariate logistic stepwise regression analysis. The assigned value was 1 in the PDA group and 0 in the control group.

## Results

### Early natural closure rate of the ductus arteriosus

**Figure 2:**
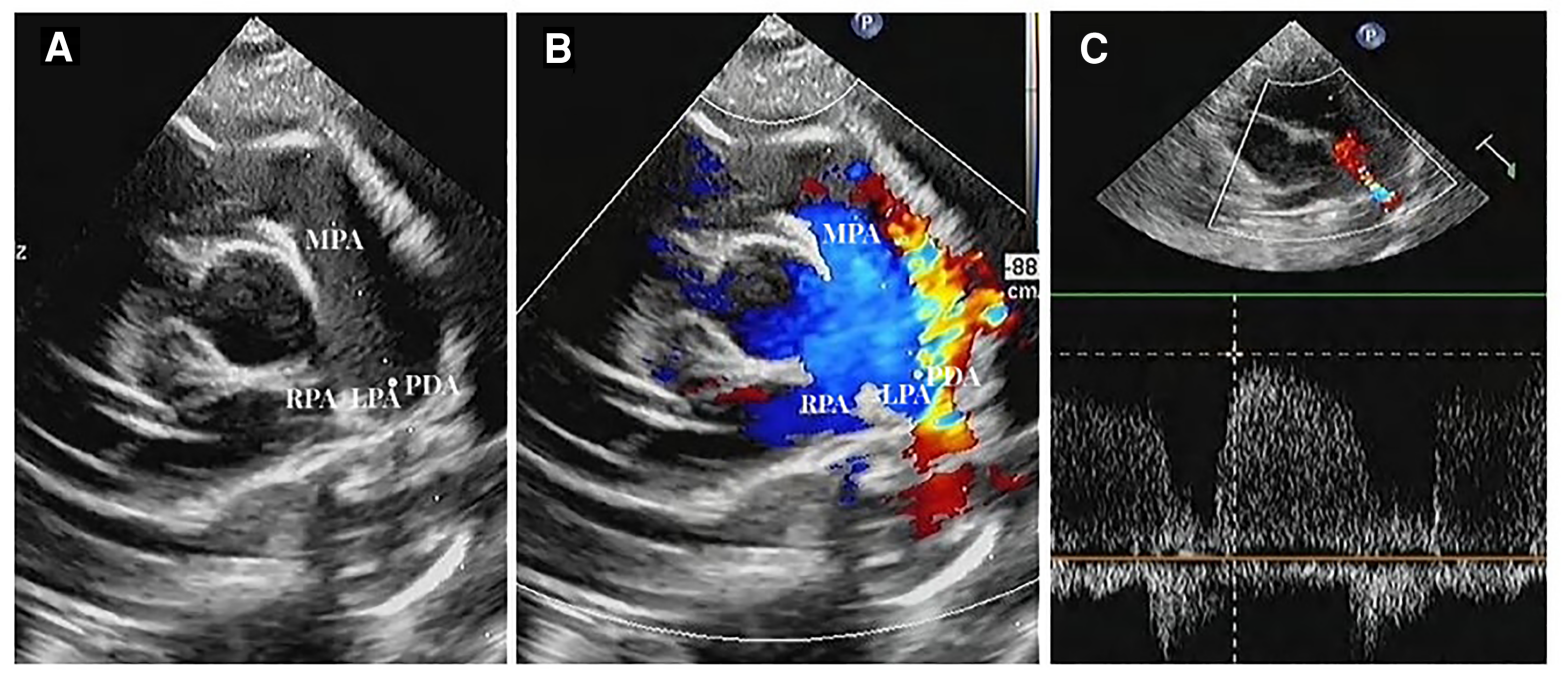
Ductus arteriosus in short axis view (**A**). Two-dimensional sonogram image; (**B**). Color Doppler sonogram image; (**C**). PW Doppler across the PDA flow (MPA, main pulmonary artery; LPA, left pulmonary artery; RPA, right pulmonary artery; PDA, unclosed ductus arteriosus).

The natural closure rate of ductus arteriosus in 222 premature infants was 50.90% (113/222) 7 days after birth, and the rate of unclosed ductus arteriosus was 49.10% (109/222). A sample of the PDA sonogram is shown in Figure 2.

### Comparison of patient characteristics

As shown in Table 1, the comparison of gestational age between the PDA group and the control group was statistically significant, and the gestational age of premature infants in the PDA group was younger than that in the control group (*P* < 0.01). This confirms that the younger the gestational age, the more prone to hsPDA. The occurrence of PDA is inversely related to GA: the younger the preterm infant, the longer the PDA naturally closes. The weight comparison analysis between the PDA group and the control group was not statistically significant. We speculate that some very low birth weight infants often have other diseases at the same time, or require early clinical intervention, and these cases were not included in our research.

**Table 1:** Statistical comparative analysis of the patient characteristics of the study population between the PDA group and the control group.

	*n*	Male %	Birthweight (g)	Gestational age (week)
PDA group	109	51.37	2,023.85 ± 730.02	31.72 ± 3.11
Control group	113	58.41	1,894.20 ± 626.90	33.30 ± 2.48
*χ*^2^/*t*		1.11	1.42	4.18
*P*-value		0.293	0.157	<0.01

### Comparison of echocardiographic parameters

The DA shunt velocity and ΔPs in the PDA group were lower than those in the control group (*P* < 0.05), while PASP in the PDA group was higher than that in the control group (*P* < 0.05) (Table 2).

**Table 2:** Comparison of echocardiographic parameters between the two groups at 48 h after birth.

	LAD/AOD	LVEF (%)	FS (%)	Tricuspid regurgitation area (mm^2^)	DA diameter (mm)	DA shunt velocity (m/s)	ΔPs (mmHg)	PASP (mmHg)	Foramen ovale width (mm)
PDA group	1.39 ± 0.28	65.57 ± 5.05	33.07 ± 3.74	31.77 ± 24.51	2.76 ± 0.76	1.30 ± 0.63	8.39 ± 7.62	45.27 ± 13.93	2.62 ± 0.67
Control group	1.46 ± 0.29	65.65 ± 5.73	33.10 ± 4.82	30.02 ± 20.01	2.59 ± 0.72	1.62 ± 0.63	12.06 ± 10.37	40.65 ± 12.84	2.78 ± 0.76
t	1.76	0.11	0.06	−0.58	−1.72	3.71	2.99	−2.57	−3.99
p	0.08	0.92	0.96	0.56	0.08	<0.01	<0.01	<0.05	0.12

LAD, left atrial end-systolic diameter; AOD, aortic root diameter; LVEF, left ventricular ejection fraction; FS, fractional shortening; ΔPs, pressure difference between descending aorta the pulmonary artery; PASP, pulmonary artery systolic pressure.

### Pearson correlation analysis of DA diameter, PASP, DA shunt velocity, and ΔPs

Ultrasonic parameters often affect each other and cannot be analyzed individually. We selected indicators that are clinically believed to have a greater mutual influence for correlation analysis, which are DA diameter, PASP, DA shunt velocity, and ΔPs of echocardiography 48 h after birth in premature infants. It was concluded from Table 3 that DA diameter was negatively correlated with shunt velocity and ΔPs, and positively correlated with PASP. DA shunt velocity was positively correlated with ΔPs and negatively correlated with PASP. ΔPs was negatively correlated with PASP. And the correlation analysis between any two of the four parameters was *P* < 0.01, showing a significant correlation.

**Table 3:** Pearson correlation analysis of ultrasonic parameters (DA diameter, PASP, DA shunt velocity, ΔPs) at 48 h after birth.

	Mean	SD	DA diameter	DA shunt velocity	ΔPs	PASP
DA diameter	2.67	0.74	1			
DA shunt velocity	1.46	0.65	−0.249**	1		
ΔPs	10.26	9.28	−2.00**	0.953**	1	
PASP	42.91	13.55	0.353**	−0.439**	−0.373**	1

** *P* < 0.01.

### Logistic stepwise regression analysis

PASP, DA shunt velocity and ΔPs were selected into Logistic stepwise regression analysis. PASP and ΔPs were not associated with early spontaneous closure of ductus arteriosus within 7 days after birth, but only the shunt velocity was associated with early spontaneous closure of ductus arteriosus within 7 days after birth (*P* = 0.049) (Table 4).

**Table 4:** Results of logistic regression analysis of ultrasonic parameters at 48 h after birth in premature infants.

	*β*	SE value	Wald *χ*^2^ value	*P* value	OR (95% CI)
DA shunt velocity	1.54	0.79	3.84	0.049 (<0.05)	1.29 (1.00–21.72)
ΔPs	−0.07	0.05	1.65	0.19	4.66 (0.84–1.04)
PASP	−0.016	0.01	1.55	0.21	0.99 (0.96–1.01)

### ROC curve results of echocardiography DA shunt velocity

**Figure 3:**
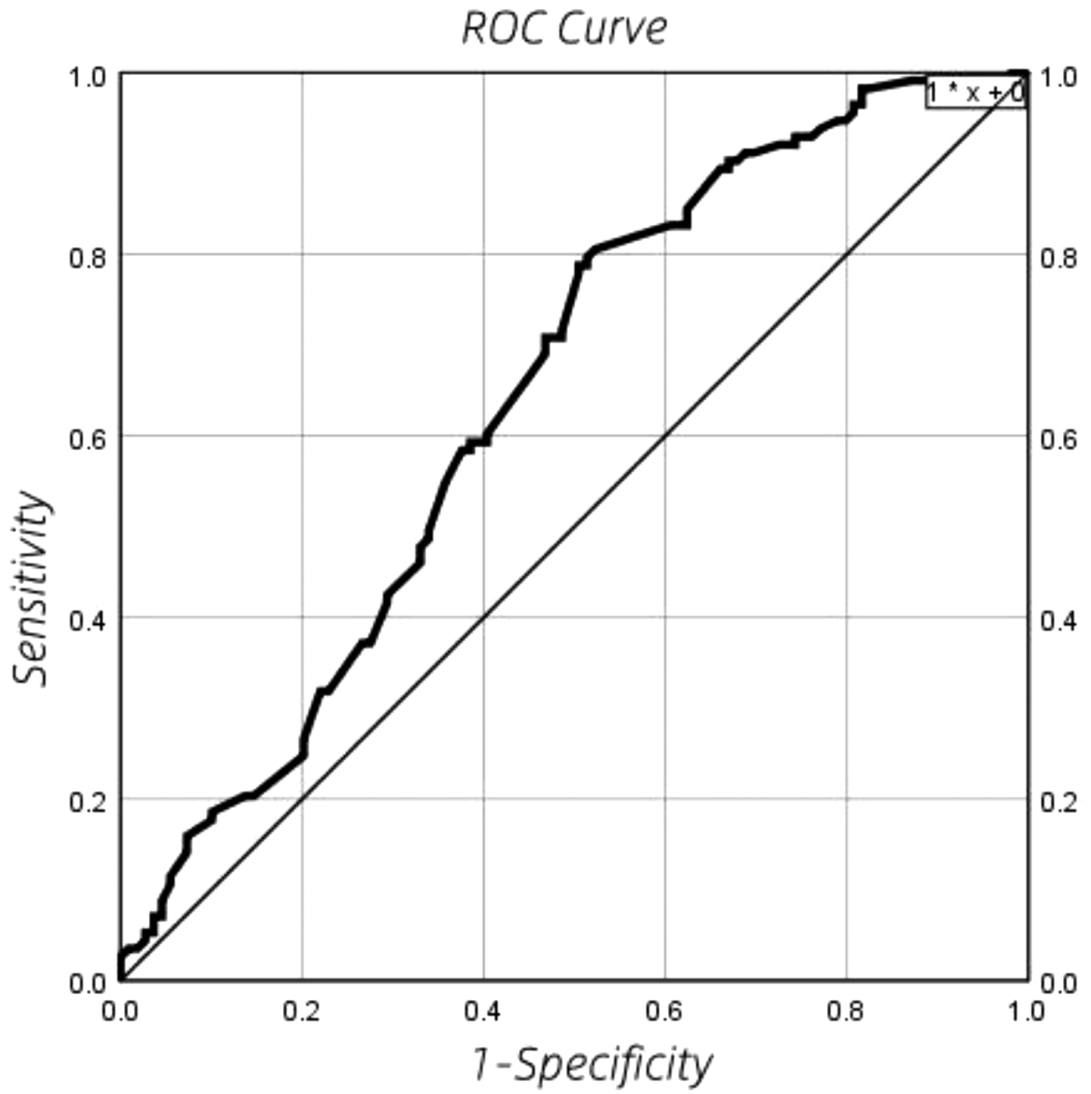
ROC curve results of echocardiography DA shunt velocity in premature infants 48 h after birth.

ROC curve is shown in Figure 3. The optimal cut-off point, sensitivity, specificity, positive likelihood ratio (sensitivity/(1-specificity), negative likelihood ratio [(1-sensitivity)/Specificity] is shown in Table 5. By drawing the ROC curve, the optimal critical point of echocardiographic DA shunt velocity in premature infants 48 h after birth was 1.165 m/s, and the corresponding specificity, sensitivity, positive and negative likelihood of the critical point predicting the early natural closure of PDA were obtained. The positive likelihood ratio and negative likelihood ratio of DA shunt velocity were 1.56 and 0.43 respectively, that is, when DA shunt velocity <1.165 m/s, the possibility of the premature infant coming from the PDA group was 1.56 times that of the control group, When DA shunt velocity >1.165 m/s, the possibility of the premature infant from the PDA group is about 0.43 times that of the control group.

**Table 5:** The optimal cut-off point, sensitivity, specificity, positive likelihood ratio [sensitivity/(1-specificity)], negative likelihood ratio [(1-sensitivity)/specificity].

Parameters	Optimal cut-off point	Specificity (%)	Sensitivity (%)	Negative likelihood ratio	Positive likelihood ratio
DA shunt velocity (m/s)	1.165	49.5	78.8	0.43	1.56

## Discussion

In this study, univariate and multivariate analysis of echocardiographic parameters of premature infants 48 h after birth were carried out, which has important clinical reference value. The continuous opening of the ductus arteriosus is affected by many factors (9). Among them, ion channel and prostaglandin induced functional closure of ductus arteriosus (3). Compared with term infants, the smooth muscle of ductus arteriosus in premature infants is relatively dysplastic, with a larger inner diameter, thinner wall and a lower sensitivity to oxygen partial pressure (10). In addition, the study of (11) has shown that gestational age and body mass are closely related to the occurrence of PDA.

From hemodynamics' perspective, LAD/AOD well reflects pulmonary blood circulation. The systolic and diastolic AOSP of premature infants is higher than PASP. This causes a portion of the aortic blood to flow to the pulmonary artery through the ductus arteriosus, resulting in increased pulmonary blood flow, and back flow to the left atrium. Consequently, the preload of the left ventricular is increased. The study of (12) has shown that the LAD/AOD ratio has certain value in predicting the ductus arteriosus. In essence, the higher the LAD/AOD value, the greater the pulmonary circulation blood flow, and the heavier the left ventricular preload. Therefore, it is less likely that the ductus arteriosus may close spontaneously. However, the comparative analysis of LAD/AOD values between the two groups in this study is not statistically significant. Our analysis of the LAD/AOD ratio is the result of left atrial enlargement, which means that the left ventricular load increases after a large portion of aortic flow is shunted, but the hemodynamic severity of the shunting effect cannot be directly evaluated. The development of left atrial enlargement takes some time. In this study, for the premature infants with PDA, the change of cardiac hemodynamics was not sufficient to cause the change of LAD/AOD significantly, so the statistical difference is not significant. The phenomenon was also reported in the study of Dix LML team and others (13).

Basically, foramen ovale closes within 1 year after birth, and the hemodynamics of most premature infants is often accompanied by the influence of PAD. Theoretically, the wider the foramen ovale, the greater the blood flow from the left atrium into the right atrium and then into the pulmonary artery through the right ventricle, resulting in higher pulmonary artery pressure and more difficult closure of the ductus arteriosus. However, the shunt flow of the foramen ovale depends not only on the width of the foramen ovale, but also on the pressure difference between the left and right atria. When the foramen ovale is open, blood from the left atrium flows into the right atrium and then enters the pulmonary artery through the right ventricle. The pulmonary blood flow increases, the pulmonary artery pressure increases, and the afterload of the right ventricle increases, causing the pressure of the right atrium to increase. At the same time, the ductus arteriosus is patent, and part of the blood from the descending aorta flows into the pulmonary artery, and the blood flow back to the left atrium increases, and the pressure of the left atrium increases. As a result, the pressure in both the left and right atria increases. In this paper, the width of the foramen ovale and the natural closure of the ductus arteriosus were not statistically significant. Our analysis may be due to the increase in left and right atrial pressure, but the pressure difference decreased. The foramen ovale disposition flow at 48 h after birth was not enough to cause significant statistical difference.

In theory, when the diameter of ductus arteriosus is small and the PASP is normal, the ductus arteriosus shunt has little effect on the cardiac function, and can often be closed spontaneously. When the diameter of the ductus arteriosus is larger, the shunting effect of the ductus arteriosus increases, and the pulmonary artery receives blood not only from the aorta, but also from the right ventricle, resulting in a significant increase in pulmonary blood flow, and increased back flow to the left atrium, leading to an increase in the left ventricular preload. Meanwhile, a large portion of the aortic flow is shunted through the ductus arteriosus, which significantly reduces the systemic blood flow. As a result, the left ventricle is thickened and enlarged to maintain the normal supply of systemic circulation. The above changes reduce the possibility of spontaneous closure of ductus arteriosus.

In this study, we observed that the smooth muscle of ductus arteriosus in premature infants with PDA is poorly developed, the arterial wall is thin, and the contractility is poor. These factors further reduce the possibility of early spontaneous closure of ductus arteriosus. According to the principle of hydrodynamics, when a fluid flows steadily in a pipe, the flow velocity is inversely proportional to the diameter of the pipe. In this study, the DA shunt velocity in the PDA group was lower than that in the control group. It may be speculated that that the lower the shunt flow speed and the wider the internal diameter of ductus arteriosus, the lower the possibility of spontaneous closure of ductus arteriosus. However, the internal diameter parameters of ductus arteriosus within 48 h in this study had no significant statistical significance in univariate analysis. This is consistent with the research reported in Jeong HY, Lee JH and others (14, 15). By drawing the ROC curve, the optimal critical point of echocardiographic DA shunt velocity in premature infants 48 h after birth was 1.165 m/s, and the corresponding specificity, sensitivity, and negative likelihood of the critical point predicting the early natural closure of PDA were obtained. The results have important guiding significance for clinical practice.

In this study, the parameters of the inner diameter of the PDA within 48 h did not have significant statistical significance in the single factor analysis. We speculate that it may also be related to the changeable shape of the PDA. The ductus arteriosus can be divided into the tube type, the funnel type, the window type, the dumbbell type, and the aneurysm type. In some cases, the PDAs are distorted, so the statistical significance of evaluating PDA with DA inner diameter parameters is not obvious.

With an existing PDA, when PASP is greater than AOSP, it will cause ductus arteriosus right-to-left shunt. In this scenario, the high PASP is controlled by drugs, which is not included in the scope of this study. When AOSP is greater than PASP, left to right shunt occurs. A left heart failure caused by left ventricular over volume load may lead to a secondary increase of PASP, and a decrease of the pressure difference between the two ends of the shunt. Therefore, it was found that the ΔPs of the PDA group were lower than that of the control group.

The study of (16) mentioned the multi-factor analysis of the early echocardiographic parameters, and considered that the ultrasonic parameters interact with each other and cannot be analyzed alone. In this study, the multivariate Logistic stepwise regression analysis showed that only the maximum shunt velocity of ductus arteriosus was related to the early natural closure of ductus arteriosus in premature infants. It is speculated that there is an interaction among the parameters of ultrasound, such as the thickness of the shunting affects, the increase of DA shunt velocity, PASP of the shunting indirectly affects the pressure of the left and right atrium, leading to the change of shunt velocity of foramen ovale, and also affects the diameter of atrioventricular and artery.

The shunt diameter is usually measured at the narrowest point of two-dimensional echocardiography. However, this may be difficult to identify manually, especially in the scenario where infants with very low birth weight would be affected by mechanical ventilation, and the ductus arteriosus in premature infants is tiny, and measurement error prone. Therefore, we will consider how to further observe the influence of DA diameter in follow-up studies. In comparison, the velocity of the ductus arteriosus shunt is less affected by manual measurement, and it can indirectly reflect the changes in DA width and the pressure difference between the two ends of the ductus arteriosus. For this reason, the maximum velocity of ductus arteriosus shunt is suggested by us as an optimal parameter in predicting whether PDA may be naturally closed.

Concerning the limitations of the study, we were only able to analyze limited number of echocardiographic parameters, because our study was a retrospective study, and data collection was limited to available ultrasound data based on our routine echocardiography. These parameters were selected as research parameters because they are easy to be popularized and repeated. However, more parameters, e.g., the lung ultrasound measurements could be added to the current parameter data set. This will be incorporated in our future work in subsequent prospective studies.

## Conclusion

The echocardiographic parameters 48 h after birth are of great value to predict the early spontaneous closure of ductus arteriosus in premature infants. In particular, the blood flow velocity in the ductus arteriosus shunt is related to the early spontaneous closure of ductus arteriosus.

## Data Availability

The raw data supporting the conclusions of this article will be made available by the authors, without undue reservation.
